# Dietary Content of Plant Ingredients and Phospholipids Affects Astaxanthin Utilization and Lipid Deposition in Atlantic Salmon (*Salmo salar* L.)

**DOI:** 10.1155/anu/3454274

**Published:** 2025-03-21

**Authors:** Trine Ytrestøyl, Bente Ruyter, Tone-Kari K. Østbye, Bjarne Hatlen, Sergey Afanasyev, Marta Bou, Grete Baeverfjord, Aleksei Krasnov

**Affiliations:** ^1^Nofima AS, Norwegian Institute of Food, Fisheries and Aquaculture Research, Tromsø, Norway; ^2^Sechenov Institute of Evolutionary Physiology and Biochemistry, Saint Petersburg, Russia

**Keywords:** astaxanthin, Atlantic salmon, digestibility, marine ingredients, phospholipids, retention

## Abstract

The effects of replacing dietary fish meal (FM) and oil (FO) with plant ingredients on absorption, metabolism, and flesh retention of astaxanthin were tested in Atlantic salmon. Phospholipid (PL) concentrates of marine (MPL) or plant origin (Soy lecithin, SoyLec) were supplemented to plant-based diets as choline sources to study potential effects on astaxanthin absorption and retention. A total of six diets were tested, three of them at high and low temperature (6 and 12°C). Lower temperature and slower growth increased retention of astaxanthin in the muscle of the marine-diet group but had no effect in the low marine-diet groups. Digestibility of astaxanthin was not affected by temperature in any of the diet groups. Sufficient PL in the diet was crucial for the digestibility of astaxanthin and lipids, but the source of phospholipid did not affect digestibility. The source of PL did have an effect on the accumulation of astaxanthin in the muscle. MPL reduced the muscle retention of astaxanthin and increased liver accumulation of the astaxanthin metabolite idoxanthin compared to plant protein (PP) diets and diets supplemented with SoyLec. PP diets also increased the deposition of lipid in liver and caused steatosis of intestine. Genes involved in formation of lipoproteins and cholesterol synthesis in the mid-intestine were downregulated in fish fed PP diets compared to a FM diet. MPL supplementation to the PP diet reduced the changes in gene expression and the steatosis in the intestine whereas adding SoyLec did not. Neither MPL nor SoyLec supplementation reduced the accumulation of lipid in liver in fish fed plant protein diets. In conclusion, the addition of MPL to a plant-based diet improved intestinal lipid transport, but not astaxanthin deposition in muscle.

## 1. Introduction

Feeds for Norwegian farmed salmon have gone through major changes in composition from a marine based diet in the early 90s to a diet with more than 70% plant ingredients [1]. In 2020, the salmon feed contained 12% fish meal (FM) and 10% fish oil (FO) [2]. This shift in ingredients of the salmon diet has resulted in reduced levels of several nutrients, such as the omega-3 fatty acids (n-3 FAs), eicosapentaenoic acid (EPA), and docosahexaenoic acid (DHA), retinol, cholesterol, and phospholipids (PL), that may potentially affect flesh pigmentation in Atlantic salmon [3–5]. The effects may be on absorptive processes in the gut but also through interference with transport and metabolic conversion of astaxanthin. Plant-based diets contain more fiber and other antinutritional factors compared to marine diets, which may be negative for the solubilization of fat and fat-soluble compounds like carotenoids and thus reduce their uptake from the gut [6–9]. High-fat diets with 70%–80% plant ingredients have been shown to increase whole body lipid retention and lipid concentrations in plasma and liver of Atlantic salmon when marine micronutrients are not added [10–12]. Accumulation of lipid droplets in the mid-intestinal mucosa has also been observed in salmonids after feeding high levels of plant proteins (PP)and plant oils (PO) [13, 14]. However, the inclusion of PL in the diet can reverse this condition [13, 15]. This indicates that PL may be a limiting factor for lipoprotein assembly in the intestine and possibly in liver of salmon fed high-PP diets. Lipoproteins are carriers of lipids, including lipid soluble pigments like carotenoids. Astaxanthin is incorporated into chylomicrons in the intestine before uptake in the enterocytes [6, 16, 17]. Dietary fatty acid composition and concentration of PL and cholesterol influence the formation of mixed micelles in the gut lumen and several studies have found effects of fatty acid composition on astaxanthin digestibility. Courtot et al. [18] found a positive correlation between the dietary content of mono- and poly-unsaturated fatty acids (MUFA and PUFA) and astaxanthin digestibility and a negative effect of diet content of saturated fatty acids (SFA). Sigholt et al. [19] found a linear decrease in digestibility of astaxanthin with increasing inclusion of palm oil, which has a high content of SFA. Several studies have also found a positive effect of a high PUFA content in the diet on astaxanthin deposition and fillet color [5, 20–24], whereas others have shown no negative effects on fillet pigmentation after feeding various PO and protein sources [25–30]. The dietary PL concentration has also been found to influence the uptake and deposition of carotenoids in salmonids [31–33]. Olsen et al. [31] found a negative effect of soy lecithin on astaxanthin concentration in plasma, whereas Salvador et al. [32] demonstrated a positive effect of soy lecithin on canthaxanthin digestibility, plasma concentration, and muscle deposition in immature rainbow trout. Lipid uptake and deposition is also affected by dietary PL. Choline and phosphatidylcholine improved lipid digestibility and reduced lipid accumulation in the intestine in salmon fed a diet with low FM content [15].

Considering the importance of salmon fillet color for consumer acceptance, surprisingly little is known about the biological factors that influence flesh color. The muscle retention of astaxanthin is low, typically between 5% and 10%, whereas the digestibility can be around 40%, depending on temperature, feed intake, and dietary factors [34–37]. Temperature may affect pigmentation of salmon through effects on digestibility, metabolism, and growth rate [36, 37]. Several studies have reported brighter fillet color at lower temperatures [38–40]. There may also be interactions between dietary fatty acid composition and temperature on utilization of astaxanthin [19].

The difference between retention and digestibility suggests that the majority of the absorbed astaxanthin is metabolized to other compounds through several pathways, and that metabolism is an important factor in determining how much astaxanthin is deposited in the salmon muscle. Astaxanthin is a precursor of retinol in fish including salmonids [41, 42], but salmonids also metabolize ketocarotenoids by a reduction of the ketogroups [43–46]. The intestine and liver are the most active organs for metabolism of astaxanthin ([47], Scmeisser et al., 2021). Recently, a study comparing red-fleshed with paler rainbow trout found an increased expression of genes involved in intestinal absorption and transport of carotenoids and lipids in the pyloric cecum from the red fillet group [48]. Some of the affected genes were CD36, phospholipid-transporting ATPase (ABCA1), fatty acid elongase 6 (ELOVL), and retinol dehydrogenase (RDH) and retinoic acid receptor. Furthermore, both the carotene cleaving enzymes BCO1 and BCO2 involved in retinol synthesis had lower expression in the muscle of the red fillet group. Helgeland et al. [49] also found higher expression of BCO1 in Atlantic salmon with paler fillet color, and the importance of metabolism in the pylorus on astaxanthin retention was also confirmed by Schmeisser et al. [50].

The present study examined the digestibility, metabolism, and deposition of astaxanthin in muscle, liver, and intestine in Atlantic salmon fed diets with marine and plant ingredients and the interaction between diet and temperature. Furthermore, the effect of adding PL from soy or from marine ingredients to a low-marine diet on pigmentation was also measured. Analysis of gene expression in liver and intestine was used to study mechanisms related to lipid and carotenoid transport and metabolism.

## 2. Methods

### 2.1. Experimental Design

**Figure 1:**
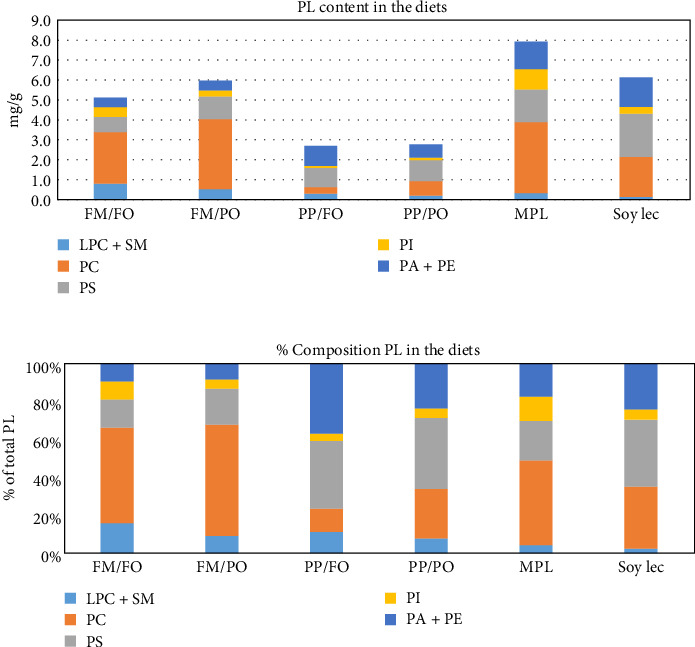
Phospholipid (PL) classes in the six diets, concentration in mg/g (A), and in % of total phospholipids (B). LPC, lysophosphatidylcholine; FM, fish meal; FO, fish oil; MPL, marine phospholipids; PA, phosphatidate; PC, phosphatidylcholine; PE, phosphatidylethanolamine; PI, phosphatidylinositol; PO, plant oil; PP, plant protein; PS, phosphatidylserine; SM, sphingomyelin; Soy Lec, soy lecitin.

The feeding experiment was carried out at the experimental station of Nofima, Sunndalsøra, Norway in a set-up described by Ytrestøyl et al. [51]. Triplicate groups of size-graded Atlantic salmon (*Salmo salar*) with an initial average weight of 197 g were fed six experimental diets with different content of marine and plant ingredients for a period of 84 days. The marine diet was high in both FM and FO. Then either FM or FO was combined with PO (rapeseed oil) or PP (soy protein concentrate and wheat gluten), respectively. In the low-marine diet both FO and FM were replaced with plant ingredients. Two diets formulated with low FM and FO content (PP/PO) were supplemented with PL from soybeans (soy lecithin, SoyLec) or of marine origin (marine phospholipid, MPL). The soy lecithin product was obtained from Denofa (Fredrikstad, Norway). The marine phospholipid was extracted from FM and produced by TripleNine (Esbjerg, Denmark). The formulation of the diets is given in Table 1. The fatty acid composition of the diets is given in Table S1. The content of the different phospholipid classes in the six diets is shown in Figure 1.

Before the experiment started, the fish were fed a commercial diet without carotenoid supplements (Skretting, Stavanger, Norway, astaxanthin content < 5 mg/kg). During the trial, fish were maintained indoors in 1 m^2^ fibreglass tanks (600 l) supplied with seawater (32 ppt), with an average temperature of 12°C. Each diet was fed in triplicate tanks. The tanks were supplied with continuous illumination during the trial. The fish were fed in 20% excess to ensure feeding to satiation. The tank outlets were passed through an excess feed collector which enabled calculation of accurate feed intake based on analyses of dry matter in the feed and the collected excess feed [52]. This provided data for estimation of astaxanthin retention in the muscle in % of the eaten amount of astaxanthin. Three of the diets (FM/FO, FM/PO, and PP/FO) were also fed at 6°C for a period of 120 days. Limited capacity was the reason why only three of the diets could be tested on both temperatures. The three diets tested on 6°C were selected to study the interactions of FM and FO with temperature.

### 2.2. Sample Collection

The salmon were anesthetized with MS-222 (metacaine, 0.1 g L^−1^) before length and weight were measured. The fish were killed by a blow to the head and samples of muscle, liver, and mid-intestine were collected for microarray analysis. Liver weight was recorded, and the visceral fat content was assessed visually by a score from 1 to 5, where 1 was the lowest, and 5 was the highest [53]. The middle section of the mid-intestine and liver was dissected out, and a small piece (1×1 mm) was frozen immediately in liquid nitrogen and kept at -80°C until analysis. The samples from muscle were taken 2 cm above the lateral line on the Norwegian quality cut (NQC). Six fish from each tank were sampled for analyses of carotenoid concentration of the muscle, liver, and intestine at the end of the feeding trial. Samples for histology were taken from the mid-intestine from six fish per tank and stored in formalin (at 12°C only). Feces were collected by stripping of the remaining fish in the tank according to Austreng et al. [54]. Blood samples were taken from the caudal vein from 6 fish per tank. The samples were centrifuged (5000 g, 10 min) and the serum was frozen (−80°C) for later analysis of carotenoids. From fish fed three of the diets (FM/FO, PP/PO, and the MPL diet) the lipoproteins were isolated from blood by ultracentrifugation as described by Aas et al. [55]. The fractions were frozen (−80°C) for later analysis of carotenoid content in the different lipoproteins as described below.

### 2.3. Analyses

#### 2.3.1. Proximate Composition of the Diets

The diets were analyzed for dry matter (DM, 105°C, 16–18 h), ash (pre-combustion on a hot plate followed by 3–4 h at 550°C), crude protein (N x6.25; semi micro Kjeldahl, Kjeltec Auto System, Tecator, Höganäs, Sweden), and crude lipid (diethyl ether extraction in a Fosstec analyzer (Tecator, Höganäs, Sweden) after HCl hydrolysis. The gross energy of the feeds was analyzed by adiabatic bomb calorimetry (Parr 1271 Bomb calorimeter, Parr, Moline IL, USA). The concentration of yttrium in the feeds and feces was determined by inductively coupled argon plasma spectrometry at Eurofins (DK). The chemical composition of the diets is given in Table 2, and the fatty acid composition of the diets is found in the Supporting Information (Table S1).

#### 2.3.2. Analysis of Carotenoids in Tissues, Feed, and Feces

The astaxanthin concentration of the feeds and fecal samples was analyzed following extraction of the carotenoids according to the procedure described by Schierle and Härdi [56]. Homogenized fillets from individual fish were thawed, and carotenoids were analyzed according to Bjerkeng et al. [34]. Two different isocratic HPLC systems were used to determine carotenoid concentrations. A H3PO4-modified silica gel column (Hibar, LiChrosorb Si 60; Merck, Darmstadt, Germany) was used to determine the geometrical E/Z isomers of astaxanthin in feed and feces. In muscle, liver, and intestine, a Spherisorb S5-CN nitrile column (PhaseSep, Queensferry, Clywd, UK) was used with 20% acetone in n-hexane as the mobile phase, to determine astaxanthin and 3′, 4′-cis and 3′, 4′-trans glycolic isomers of idoxanthin. Standards of known concentration were prepared from crystalline all-E-astaxanthin (Hoffmann-La Roche Ltd, Basel, Switzerland), and the concentration of the standard solution was measured spectrophotometrically (UV-260, Shimadzu, Japan) using molar absorptivity E1%, 1cm = 2100 at absorbance maximum (*λ* max = 470 nm) in n-hexane containing 4.5% chloroform. The percentages of the different isomers were calculated from chromatogram areas and corrected for differences in extinction coefficients (E1%, 1 cm) [57, 58].

#### 2.3.3. Analysis of Liver Lipid Class Composition

Total lipids were extracted from liver using the method described by Folch et al. [59]. The lipid class composition of the liver (free fatty acids, (FFA), monoacylglycerol/diacylglycerol (MAG/DAG), triacylglycerol (TAG), and PL was analyzed by thin-layer chromatography (TLC) as described by Todorčević et al. [60].

#### 2.3.4. Histology of Mid-Intestine

The mid-intestine from 4 to 6 fish per diet was evaluated histologically at the end of the trial at 12°C. Samples of mid intestine were fixed in 4% buffered formalin and stored at 4°C until processing with standard histological techniques and staining with hematoxylin and eosin. A total of 34 samples were evaluated by light microscopy, blinded and randomized. The samples were evaluated with particular focus on excessive lipid accumulation in enterocytes [61, 62]. Lipid accumulation was, when present, classified as mild, moderate, or severe (classification adapted from [62]).

#### 2.3.5. Microarray Analysis

RNA was extracted with Biomek 4000 robot using Agencourt RNAdvance Tissue kit (Beckman Coulter), quality was assessed with Agilent Bioanalyzer 2100, RNA 6000 n kit (RIN > 8). Nofima's genome-wide Atlantic salmon microarray Salgeno with 44 k 60-mer oligonucleotide probes was used. The platform was annotated with bioinformatics package STARS [63]. Analyses were performed in the intestine, liver, and skeletal muscle (*n* = 5) of four groups at 12°C: FM/FO, PP/PO, MPL, and SoyLec. In total, 60 microarrays were used. Microarrays were manufactured by Agilent Technologies, and the reagents and equipment were purchased from the same provider. RNA amplification and labeling were performed with a One-Color Quick Amp Labeling Kit, and a Gene Expression Hybridization kit was used for fragmentation of labeled RNA. Total RNA input for each reaction was 500 ng. After overnight hybridization in an oven (17 h, 65°C, rotation speed 0.01 g), arrays were washed with Gene Expression Wash Buffers 1 and 2 and scanned with Agilent scanner.

### 2.4. Calculations and Statistical Analysis

Specific growth rate (% day^−1^) was calculated as

SGR = (ln BW2 – ln BW1)×100/d (BW = bodyweight, *d* = number of days)

The thermal growth coefficient, TGC, was calculated as

TGC = 1000×(BW2 ^1/3^ – BW1^1/3^) × (number of day degrees)^−1^

The feed conversion ratio (FCR) was calculated as: feed eaten (kg)/weight gain (kg).

Hepato-somatic index (HSI) was calculated from BW and liver weight (WL):

HSI = 100×WL/BW

Individual weight and fork length were recorded to calculate condition factor (CF):

CF = BW×L^−3^ x100

Apparent digestibility ADC = 100×[1- ((X _feces_/Y _feces_)/ (X _feed_/*Y*_feed_))]

*X* = concentration (mg kg^−1^) of astaxanthin/fat and *Y* = concentration of yttrium oxide

The retention of astaxanthin in the muscle was calculated as:

Retention efficiency (%) = 100×(*W*_mu_/BW)×[(BW_F_×Ax_F_) − (BW_I_×Ax_I_)/FI×FCR×Ax_feed_]

where *A*_I_ and Ax_F_ are the initial and final astaxanthin concentrations in the flesh, respectively, BW_I_ and BW_F_ are the initial and final BW and *W*_mu_ is the weight of the flesh, which was assumed to comprise 60% of the total body weight in Atlantic salmon.

Statistical analysis were performed in SAS Jmp. A one-way ANOVA with diet as fixed factor was performed at each temperature treatment. A 2-way ANOVA was used to study effects of temperature and diet and interactions. A factorial analysis was performed for the FM/FO, FM/PO, PP/FO and PP/PO diet groups at 12°C and for the FM/FO, FM/PO and PP/FO diet groups at 6°C. Differences between treatments were ranged with Tukey's multiple range test.

Response variables given in percent were arcsin transformed before analysis by ANOVA. *p*-values < 0.05 were considered significant.

Analyses of microarray data were carried out with STARS. Global normalization was performed by equalizing the mean intensities of all microarrays. Next, the individual values for each feature were divided to the mean value of all samples producing expression ratios (ER). The log2-ER were calculated and normalized with the locally weighted non-linear regression (Lowess). Differentially expressed genes (DEG) were selected by the following criteria: expression ratio > 1.75-fold and *p*< 0.05.

## 3. Results

### 3.1. Fish Growth and Feed Utilization

Final BW, growth rates (SGR, TGC), and condition factor (CF) are given in Table 3. Fish fed diets with low FM (PP/PO and PP/FO) had lower growth rates compared to the other diets. Replacing FO with rapeseed oil did not affect growth in either PP or FM diets. Adding PL to the low-marine diet (PP/PO) improved growth, and there was no significant difference in growth between fish fed the diet with marine phospholipids (MPL) and the diets with FM (FM/FO and FM/PO). Adding soy lecithin (SoyLec) also improved growth, but the growth rate in this group was lower than in groups fed FM diets. The FCR was higher for fish fed diets with FM compared to the diets with PP (*p*< 0.0001), except the diet with MPL which did not differ from the FM/FO diet.

Fish on 6°C had lower SGR and final BW but higher TGC compared to fish fed the same diets on 12°C. As on 12°C, fish fed the low FM diet (PP/FO diet) had lower growth compared to fish fed the high marine diet (FM/FO diet) and the FM/PO diet. FCR was similar on the two temperatures.

### 3.2. Lipid Accumulation in Liver and Intestine

**Figure 2:**
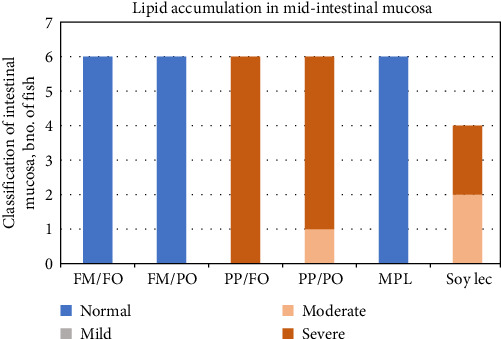
Histological evaluation of lipid accumulation in mucosa of mid-intestine. Scoring system adapted from Hansen et al. [62] (normal-mild-moderate-severe). Data were obtained in fish reared at 12°C. Bars represent no of fish per category by diet (N = 4-6 per diet). FM, fish meal; FO, fish oil; MPL, marine phospholipids; PO, plant oil; PP, plant protein; Soy Lec, soy lecitin.

**Figure 3:**
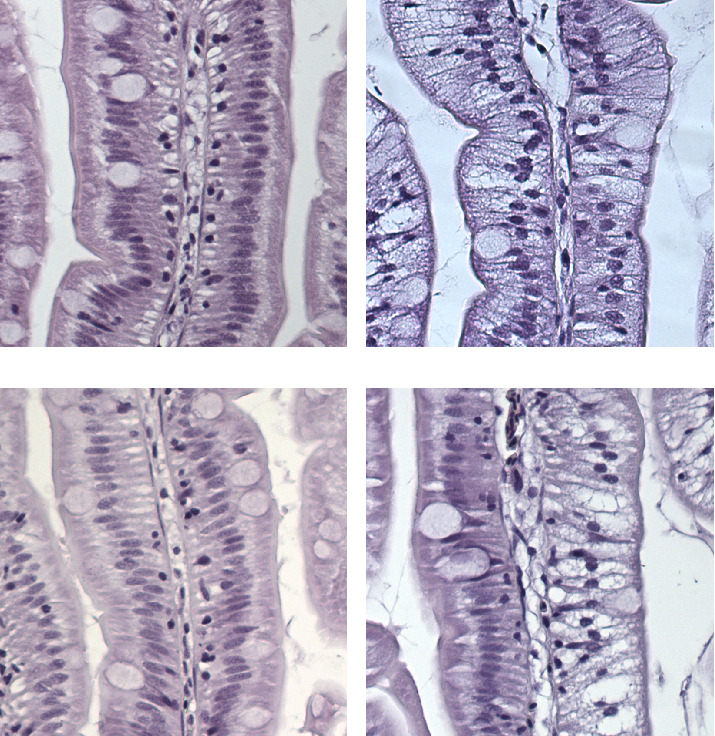
Histology of mid-intestine in salmon fed different diets. (A) FF/FO, normal vacuolization, (B) PP/PO, lipid accumulation in mucosa (severe), (C) MPL, normal vacuolization, (D) Soy lecithin, with partly normal mucosa (moderate).

Histology of the mid-intestine revealed the presence of lipid accumulation in enterocytes in mucosa, visible as ample amounts of clear vacuoles in the cytoplasm. Salmon that received feeds with FM as a protein source had normal intestines whereas fish that received fish PP diets were severely affected (Figure 2), independent of oil source (FO, PO). Supplementation with marine phospholipid (MPL) to a PP diet alleviated the symptoms completely, resulting in intestines with normal vacuolization. Supplementation with soy lecithin, on the other hand, reduced the extent of lipid vacuolization to some degree, but the deviation from normal morphology was still prominent. Histology from selected diets is shown in Figure 3. No samples from fish reared at 6° were examined.

The mean visceral fat score (1–5) was highest in fish fed diets with FM or MPL (Table 3) and showed strong positive correlation with body weight (*y* = 0.0063x + 1.97, R2 = 0.90, *p*< 0.0001). There was no effect of temperature on scores for visceral fat.

**Figure 4:**
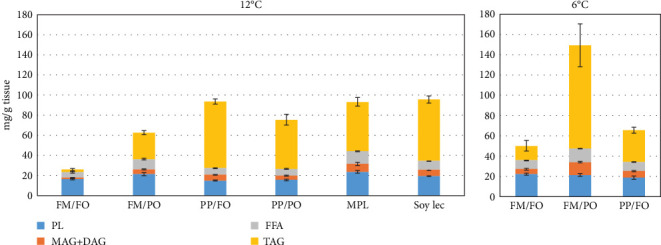
Distribution of lipid classes in liver of fish fed the different diets at 12°C. Values are means per treatment ± SEM. (*N* = 3). FFA, free fatty acids; FM, fish meal; FO, fish oil; MAG+DAG, monoacyl- and diacylglycerols; MPL, marine phospholipids; PL, phospholipids; PO, plant oil; PP, plant protein; Soy Lec, soy lecitin; TAG, triacylglycerols.

The hepatosomatic index (HSI) was lower in fish fed the FM/FO, FM/PO, and MPL diets compared to the other diet treatments (*p*< 0.0001, Table 3). There was no effect of dietary oil source on HSI, but temperature had a significant effect on liver size, HSI was higher at 6°C than at 12°C (*p*< 0.0001). There was also a significant effect of diet on the liver fat content, both at 6 and 12°C (*p*< 0.0001). The lowest fat content was found in the liver of salmon fed the marine-diet FM/FO at both temperatures (Table 3). Higher fat content was found in livers of salmon fed diets with plant protein, but rapeseed oil also led to an increase in liver fat content, particularly at 6°C (*p*< 0.0001). The lipid composition of the liver was also affected by diet (Figure 4). Fish fed the marine diet had a much lower content of triacylglycerols (TAG) than fish fed the other diets (*p*< 0.0001) and a lower content of monoacyl- (MAG) and diacylglycerols (DAG). The highest phospholipid (PL) content in the liver was found in fish fed the FM/PO diet and the diets supplemented with PL (MPL and SoyLec). At 6°C, there were no differences in PL in liver between the diet groups, but there were higher MAG/DAG and TAG in fish fed with FM and rapeseed oil (FM/PO) compared to the other two diets (Figure 4). The fatty acid composition in the liver at 6 and 12°C is shown in the Supporting Information (Table S2). Diet fatty acid composition had the strongest effect on the liver fatty acid composition, with higher concentration of the N-3 fatty acids, including EPA and DHA in liver of fish fed diet with FM and FO, and higher concentration of N-6 fatty acids in fish fed diets with rapeseed oil. But temperature also had a significant effect on the concentration of some fatty acids. Short chain saturated or monosaturated fatty acids increased in concentration at 6°C, whereas N-3 fatty acids were more abundant at 12°C. The concentration of N-6 fatty acids was not affected by temperature. There were also many interactions between diet and temperature, indicating that the response to temperature was dependent on the diet composition.

### 3.3. Digestibility of Lipid and Astaxanthin

**Figure 5:**
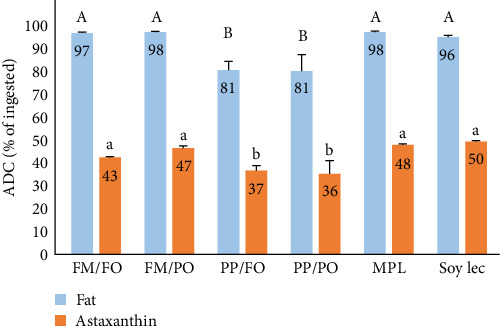
Apparent digestibility (ADC) of astaxanthin and fat of the six diets at 12°C. Values are means per treatment (*N* = 3). Differences between diets are indicated by different letters. FM, fish meal; FO, fish oil; PO, plant oil; PP, plant protein; MPL, marine phospholipids; Soy Lec, soy lecithin.

The digestibility of both astaxanthin and fat was reduced when salmon was fed diets with low fishmeal content compared to diets with fishmeal (Figure 5). Replacing FO with rapeseed oil did not affect the digestibility of astaxanthin and fat at 12°C. Addition of extra phospholipid to low FM diets, increased the apparent digestibility (ADC) of astaxanthin and fat to the levels found in diets with FM. The ADC increased proportionally to the content of phospholipid in the feed in a linear fashion (*p*< 0.001, *R*^2^ = 0.90). There was no significant effect of temperature on astaxanthin digestibility, but PO had a positive effect on digestibility of astaxanthin at 6°C (*p*< 0.01). The ADCs in the FM/FO, FM/PO, and the PM/PO diets were 40%, 47%, and 35% at 6°C, respectively.

### 3.4. Uptake and Metabolism of Astaxanthin in Serum and Tissues

#### 3.4.1. Uptake in Serum and Lipoproteins

Astaxanthin in serum was measured only in fish at 12°C and the concentration was lower in serum of salmon fed the PP/PO and MPL diets (Table 4, *p*< 0.01) than in the other diets. Adding MPL also resulted in a higher serum concentration of the astaxanthin metabolite idoxanthin compared to the other diets. Fish fed the low-marine diet (PP/PO) and the diet with plant protein and FO (PP/FO) had the lowest serum concentrations of idoxanthin. Oil source did not have an effect on serum idoxanthin, whereas FM in the diet gave higher idoxanthin concentrations in serum (*p*< 0.01).

**Figure 6:**
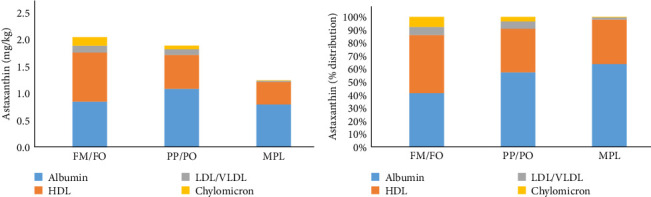
Astaxanthin in lipoprotein fractions, (A) concentration of astaxanthin in lipoprotein fractions in plasma (mg/kg), (B) % distribution of astaxanthin in the different lipoprotein fractions. FM, fish meal; FO, fish oil; PO, plant oil; PP, plant protein; MPL, marine phospholipids.

The distribution of carotenoids among plasma lipoprotein fractions was investigated in fish fed three of the diets at 12°C; FM/FO, PP/PO and the diet with MPL. The fish fed the diet with MPL had lower astaxanthin concentrations in the HDL, LDL/VLDL, and chylomicron fractions compared to the other two diet groups (Figure 6A). A larger proportion of the astaxanthin was found in lipoproteins (chylomicrons, LDL/VLDL, and HDL) in salmon fed the marine diet compared to salmon fed the low-marine diets (Figure 6B, *p*< 0.01). In salmon fed the marine diet close to 60% of the total, astaxanthin was found in the lipoprotein fractions, whereas in the salmon fed the low-marine diet, around 40% of the astaxanthin was found in the lipoprotein fraction. Addition of MPL to a low-marine diet did not change the distribution of astaxanthin between lipoproteins and albumin, it was similar as for the low-marine diet.

#### 3.4.2. Carotenoids in Intestine and Liver

There were effects of both diet and temperature on the concentration of astaxanthin and metabolites in the mid-intestine (Tables 4 and 5). At 12°C, the concentration of astaxanthin was highest in fish fed the diet with plant protein and FO (PP/FO) and lowest in the low-marine diet (PP/PO) (Table 4). Thus, at 12°C there was a significant effect of oil source when the protein source was plant protein, the astaxanthin concentration was higher in the intestine of fish fed a diet with FO (*p*< 0.01). At 6°C, the highest concentration of astaxanthin in the intestine was also found in fish fed the PP/FO diet (Table 5). The astaxanthin concentration in the intestine was slightly higher at 6°C than at 12°C (*p*< 0.05). The concentration of idoxanthin in the intestine was significantly higher at 6°C than at 12°C (*p*< 0.001). There was also a dietary effect on the concentration of idoxanthin in the intestine, with salmon fed diets with FM and MPL having higher concentrations of the metabolite (*p*< 0.001). In addition to astaxanthin and idoxanthin, two other carotenoids were detected in the intestine, one with a retention time of about 3 min and one with a retention time of 3.9 min. The concentration of the latter metabolite and idoxanthin was positively correlated (*y* = 0.23x + 0.074, R^2^ = 0.71). The identity of the two other carotenoids is unknown.

At 12°C, the content of astaxanthin in the liver was much lower in fish fed a low-marine diet (0.57 mg/kg) compared with the other diets which had similar astaxanthin levels between 1.2–1.6 mg/kg (Table 4, *p*< 0.0001). The sum of carotenoids was highest in the MPL diet due to a high content of idoxanthin (33% of total carotenoids). The concentration of idoxanthin was lowest in livers of fish fed the low-marine diet (5% of total carotenoids) and higher in fish fed FM diets (*p*< 0.05).

Temperature affected the deposition of astaxanthin and metabolites in liver and intestine. Higher concentrations of astaxanthin and idoxanthin and other metabolites were found in all three diet groups at 6°C compared to 12°C (*p*< 0.001). At 6°C, the highest content of astaxanthin was found in the liver of fish fed the marine diet (*p*< 0.05). Both FM and PO increased the liver concentration of idoxanthin (*p*< 0.01), and fish fed the PP/FO diet had the lowest liver idoxanthin concentration at 6°C (11% of total carotenoids). Adding rapeseed oil to a FM diet increased the content of idoxanthin in the liver at 6°C (*p*< 0.001), so that fish fed the FM/PO diet had the highest liver idoxanthin concentration at 6°C (40% of total carotenoids). In contrast, there was no effect of oil source at 12°C on the liver concentration of idoxanthin. There was no correlation between the content of astaxanthin and the fat content in the liver (R^2^ = 0.001) at either temperature.

#### 3.4.3. Deposition of Carotenoids in Muscle

The concentration of astaxanthin in the muscle was affected both by diet and temperature (*p*< 0.0001, Table 4 and 5). At 12°C, salmon fed the low-marine diet supplemented with soy lecithin (SoyLec) had the highest astaxanthin concentration in the muscle (3.1 mg/kg) and fish fed the low-marine diet supplemented with MPL had the lowest astaxanthin concentration in the muscle (1.9 mg/kg). The FM and the low-marine diets gave similar muscle astaxanthin concentrations (2.3–2.4 mg/kg). Replacing FO with rapeseed oil did not affect muscle astaxanthin concentration at 12°C. Fish fed the diet with MPL had the highest muscle concentration of idoxanthin (0.65 mg/kg) whereas the lowest concentrations were found in salmon fed the PP/PO diet and the PP/FO diet (7% of total carotenoids). The diets with FM gave intermediate muscle idoxanthin concentrations. Thus, FM in the diet gave higher muscle idoxanthin concentrations, and adding MPL increased idoxanthin even more. In salmon fed FM diets, idoxanthin made up 15%–18% of muscle carotenoids, and in salmon fed the MPL diet, idoxanthin made up 26% of total carotenoid. There was a positive linear correlation between the content of idoxanthin in muscle and the content of phospholipid in the feed (R^2^ = 0.85).

The muscle retention (% of intake) of astaxanthin was highest in the diet with SoyLec (7.9%) followed by the PP/FO diet where the retention of astaxanthin was 7.7% (Table 4). The lowest retention of astaxanthin in the muscle was found in salmon fed the MPL diet (4.2%), but also the diets with FM had low retention values (5.7%–5.9%). For diets with either PO (FM/PO) or plant protein (PP/FO), the astaxanthin retention was unaffected by temperature (Table 4 and 5), while for the FM/FO diet the retention of astaxanthin was significantly higher at 6°C (7.2%) than at 12°C (5.9%, *p*< 0.001). The lowest retention at 6°C was found in fish fed the FM/PO diet (5.7%). At 12°C, no negative effects on astaxanthin retention were found when FO was replaced by rapeseed oil in a high FM diet. However, for the FM/FO diet, the retention of astaxanthin was 1.3% higher at 6° than at 12°C (*p*< 0.001).

### 3.5. Microarray

The analysis of gene expression was performed on liver and mid intestine from fish fed four diets at 12°. Results for the PP/PO, SoyLec, and MPL diets are expressed as the difference in relation to the marine diet (FM/FO). Clear effects of diet on gene expression were found in both tissues. Fish fed the diet supplemented with MPL were most similar to the fish fed the FM/FO diet and had the fewest number of differentially expressed genes (DEG); 157 in intestine and 151 in liver. Fish fed the Soy Lec diet had 323 and 160 DEGs in intestine and liver, respectively, whereas fish fed the low marine PP/PO diet had the highest number of DEGs, 545 and 845 in intestine and liver, respectively. Gene sequences can be found in the Supporting Information (S3).

#### 3.5.1. Intestine

**Figure 7:**
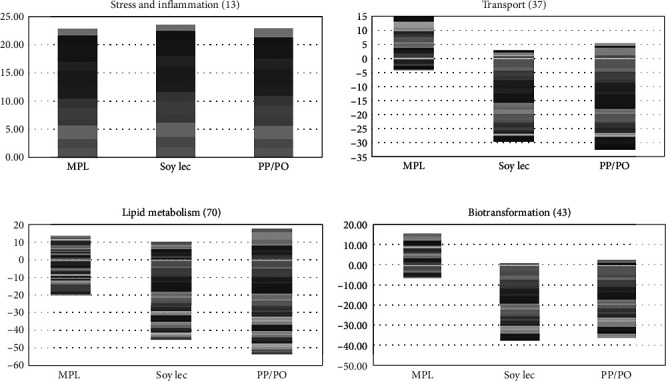
Gene expression in mid-intestine. Genes are grouped by functions and each band represents one gene, numbers of DEG are indicated between brackets. Data are log2-Expression Ratio to control (FM/FO). FM, fish meal; FO, fish oil; PO, plant oil; PP, plant protein; MPL, marine phospholipids; Soy Lec, soy lecitin.

In the mid-intestine, low-marine diets led to increased expression of genes induced by inflammation and stress, and a down-regulation of genes associated with lipid metabolism and biotransformation (Figure 7). The addition of MPL to the low-marine diet reduced part of the changes, and there were only minor differences between MPL and the marine diet.

**Figure 8:**
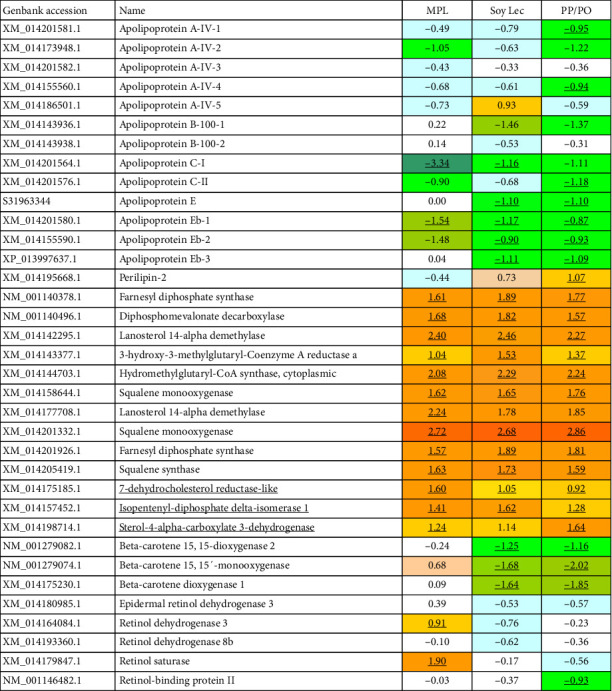
Expression of genes related to steroid metabolism, lipoprotein transport, and deposition in mid intestine. Data are log2-Expression Ratio to control (FM/FO), differential expression is indicated with bold underlined. FM, fish meal; FO, fish oil; PO, plant oil; PP, plant protein; MPL, marine phospholipids; Soy Lec, soy lecitin.

Compared to the marine diet group, *perilipin-2*, which coordinates lipid homeostasis in the gut by modulating lipid uptake capacity and transport in enterocytes, was upregulated in the SoyLec and PP/PO diet groups (Figure 7), but not in the MPL group. The intestine of fish fed the MPL and marine diets had similar expression of genes encoding apolipoproteins whereas these genes were significantly downregulated in the gut of the SoyLec and PP/PO groups, which can indicate reduced synthesis of chylomicrons. Upregulation of genes involved in the biosynthesis of steroids and terpenoids was also found in the three low-marine diets (Figure 7). Increased expression of these genes in Atlantic salmon has been consistently observed at low levels of dietary omega-3 fatty acids [64]. Low-marine diets led to a down-regulation of genes involved in the synthesis and transport of retinoids, such as *beta-carotene 15*,*15-dioxygenase* which cleaves beta-carotene into retinal, the first step in the synthesis of vitamin A in mammals. In the low-marine diet without added phospholipid, there was also a down-regulation of *retinol binding protein II*. In the diet supplemented with MPL, there were only minor effects on genes involved in retinoid synthesis and transport, except for upregulation of *retinol saturase* and *retinol dehydrogenase* which was not seen with the other low-marine diets (Figure 8).

Compared to the marine diet, genes related to synthesis of apolipoproteins were downregulated in fish fed low-marine diets (Figure 8). *Apo AIV* was downregulated in all plant diet groups, but more in the PP/PO diet, which could indicate reduced assembly of chylomicrons and lipoproteins in the intestine. Genes involved in synthesis of sterols, like cholesterol, were upregulated in all plant diet groups compared to the marine diet, suggesting increased cholesterol synthesis in fish fed in plant-based diets which are low in cholesterol.

#### 3.5.2. Liver

**Figure 9:**
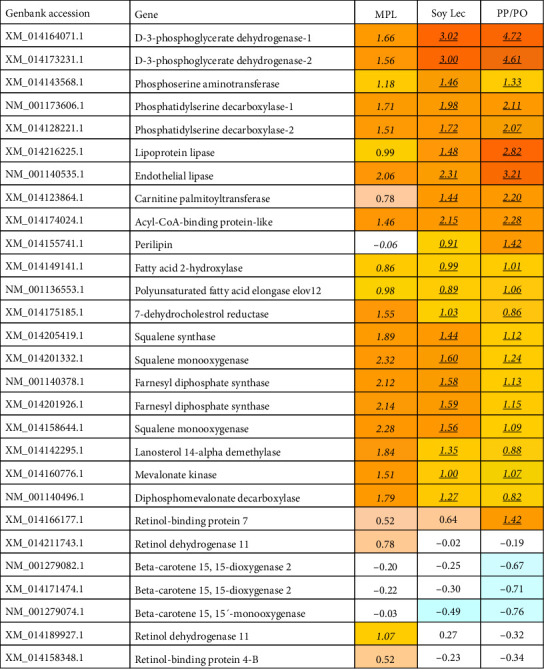
Expression of genes involved in metabolism of serine, lipids, and carotenoids in liver. Data are log2-Expression Ratio to control (FM/FO). Differential expression is indicated with bold underlined. FM, fish meal; FO, fish oil; PO, plant oil; PP, plant protein; MPL, marine phospholipids; Soy Lec, soy lecithin.

In the liver, low-marine diets led to upregulation of genes linked to amino acid metabolism, especially to the synthesis of serine (Figure 9). The enzymes *D-3 phosphoglycerate dehydrogenase 1 and 2* that are involved in the *de novo* synthesis of L-serine, were upregulated in liver of fish fed diets with plant protein and PO compared to a marine diet. The expression was highest in the PP/PO diet group and intermediate in the SoyLec, but also fish fed the MPL diet had increased expression of these genes, although not significant. The concentration of L-serine has been associated with ameliorating alcoholic fatty liver in mice, and fatty liver was found in all diet groups fed plant protein and oil in this study, only fish fed FM had normal fat content in the liver.

A panel of genes involved in lipid metabolism were also upregulated (Figure 9). Expression of *perilipin* was not different between the FM/FO diet group and the MPL group but was significantly upregulated in the PP/PO and the SoyLec diet groups. Like the intestine, liver of fish fed the low-marine diets showed signs of omega-3 deficiency, indicated by increased expression of genes associated with the synthesis of steroids and terpenoids, and the expression was higher in the MPL diet group than in the SoyLec and PP/PO groups. Low-marine diets showed an upregulation of *retinol-binding protein 7* in the liver to varying degrees compared to the marine diet. In contrast to the other diet groups, fish fed the MPL diet also had upregulation of *retinol dehydrogenase i*n liver.

**Figure 10:**
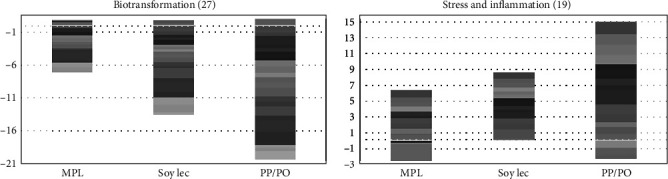
Dietary effects on genes involved in responses to stress and inflammation and xenobiotic metabolism in liver. Data are log2-Expression Ratio to control (FM/FO). Each band represents one gene, and numbers of DEG are indicated between brackets. FM, fish meal; FO, fish oil; PO, plant oil; PP, plant protein; MPL, marine phospholipids; Soy Lec, soy lecithin.

Stimulation of responses to stress and inflammation was found in parallel with suppression of xenobiotic metabolism and biotransformation, especially in fish fed the PP/PO diet (Figure 10).

## 4. Discussion

The mid intestine of salmon fed a low-marine diet displayed excess lipid accumulation in intestinal mucosa, (steatosis), as described previously in several studies [15, 61, 65, 66]. The accumulation of fat in the intestine was accompanied by reduced feed intake and growth. The accumulation of fat in vacuoles in the intestinal mucosa indicated that the transport of fat through the intestinal cells was inhibited when the content of PL in the diet was low. The intestine of fish fed the MPL diet had a relatively similar expression of several genes involved in formation of apolipoproteins and lipoprotein as the FM dietary groups, and these diet groups also had a normal vacuolization of the intestinal mucosa. Perilipin-2 is a key protein that coordinates lipid homeostasis in the gut by modulating lipid uptake capacity and transport in enterocytes. The intestine of fish fed the MPL diet had similar expression of genes related to several apolipoproteins/lipoprotein formation as the marine diet group. These genes were significantly downregulated in the gut of the SoyLec and PP/PO groups, indicating possible shortage of apoproteins required to form chylomicrons and HDL. Thus, reduced lipoprotein formation for the transport of fat out of the intestine is probably the reason for the accumulation of fat in the intestine in the present study. The addition of MPL and SoyLec to a low-marine diet had different effects on fat accumulation in the intestine. While MPL normalized the intestine and prevented steatosis, the addition of the corresponding level of SoyLec resulted only in a slight improvement of the condition of the mucosa. However, a higher dietary concentration of soy lecithin might have given a further reduction in fat accumulation. In an experiment with salmon fry the addition of PL from krill and soy lecithin both improved growth and reduced steatosis in the intestine, but soy lecithin had to be added at a higher inclusion level (3.5%) to achieve the same effect as 2.5% krill PL [67]. Similarly, Hansen et al. [62] demonstrated a dose–response relationship between dietary choline and lipid accumulation in mucosa. The results from the present study support the findings of Krogdahl et al. [15], showing that addition of choline and phosphatidylcholine to a low FM diet induces genes required for lipoprotein assembly in the intestine and reduces steatosis.

Krogdahl et al. [15] also found that the phosphatidylcholine supplemented diet had higher lipid digestibility than the low FM diet. The same result was obtained in the present study, the digestibility of both fat and astaxanthin was reduced in the low FM groups compared with the high FM groups. When the low FM diet was supplemented with phospholipid of marine origin or soy lecithin, the digestibility of both astaxanthin and fat was improved and the digestibility of astaxanthin and fat was correlated with the concentration of PL in the feed. This indicates that PL is important for the solubility of astaxanthin and fat in micelles in the intestinal lumen, which is essential for efficient uptake of astaxanthin into the intestinal cells. The content of PL in the intestinal lumen may have affected the number of micelles formed in the intestine, and perhaps also the size of the micelles. The accumulation of fat in the intestine may have contributed to the reduction in ADC, as the fat stores in the enterocytes become overloaded, the uptake and transport of fat through the enterocytes is consequently reduced. Only a few studies have addressed the effects of PL on the digestibility and serum transport and muscle deposition of carotenoids in salmonids [31–33] and the results are conflicting. Olsen et al. [31] found a negative effect of soy lecithin on astaxanthin concentration in plasma, whereas Salvador et al. [32] demonstrated a positive effect of soy lecithin on canthaxanthin digestibility, plasma concentration, and muscle deposition in immature rainbow trout. It was also shown that PL combined with FO in the diet had a greater effect than PL alone, the highest apparent canthaxanthin digestibility coefficient was obtained by a mixture of lecithin and FO. In a later study, no effect of increasing doses of soy PL on flesh canthaxanthin concentration was observed, although the concentration of canthaxanthin in all lipoprotein fractions increased with dietary PL concentration [33]. All of the studies mentioned above used far higher dietary concentrations of FM (38%–58%) compared to the diets with added PL in the present study (7.5%), which makes comparison with the former studies difficult. Olsen et al. [31] used a much higher diet concentration of soy lecithin (10%), than Salvador et al. [32], who used a comparable concentration of soy lecithin as in the present study (1.6% of the diet) and found positive effects on digestibility and pigmentation. Thus, it could be that a high diet concentration of soy lecithin could be negative for pigmentation. However, Olsen et al. [31] did not measure muscle deposition of astaxanthin, so it is not possible to conclude whether there was any effect on flesh pigmentation.

Fish from all diet groups except the high marine diet, developed fatty liver due to accumulation of triacylglycerols, as shown in several previous studies [68. 69], suggesting independent effects of oil and protein source. The addition of MPL to low FM diets did not result in a reduction in fat deposition in liver as in intestine, suggesting that factors in fishmeal other than PL affect liver fat levels. Ruyter et al. [70] found that salmon fed a FM-based diet with 100% soybean oil diet had higher accumulation of fat in the liver than fish fed a 100% FO diet at 5°C while there was no difference in intestinal fat content. Fat accumulation was also higher in both liver and intestine at 5°C than at 12°C [70]. Lipid accumulation in liver at prolonged exposure to low temperatures has been found in several fish species, including salmon [71–73]. Low levels of EPA and DHA in the diet also promote liver lipid accumulation [61], particularly at low water temperatures [74]. Increased fat accumulation in liver and intestine can be a result of disturbances of several metabolic pathways; increased lipogenesis, inhibition of fatty acid oxidation, increased uptake, or reduced secretion of lipids. Upregulation of genes involved in the biosynthesis of steroids and terpenoids in the low-marine diets is a hallmark of low-marine diets [64]. Perilipin 2 has also been shown to promote obesity and progressive fatty liver disease in mouse experiments [75]. In other human and mouse studies, the hepatic perilipin 2 levels have been correlated to the degree of hepatosteatosis in several types of liver diseases [76]. Compared to the high marine group, gene expression of *perilipin-2* was upregulated in the SoyLec and PP/PO diet groups in the present study. Recent studies have shown an association between serine metabolism and fat accumulation in the liver. Mice fed a diet enriched with L-serine ameliorated alcoholic fatty liver by accelerating L-serine-dependent homocysteine metabolism [77]. A genome-scale metabolic modeling of human hepatocytes revealed a link between serine deficiency in patients with non-alcoholic liver disease [78]. This is in line with our findings showing upregulation of genes in serine metabolism in all PP groups compared to the high marine diet.

The large difference in muscle retention of astaxanthin between the MPL diet group (4.2%) and the SoyLec diet which had the highest retention (7.9%) was surprising. Fish fed the two diets had similar final BW, and the digestibility of astaxanthin was also similar for both diets. The total concentrations of PL were slightly higher in the MPL diet than in the SoyLec diet due to higher levels of phosphatidylcholine and phosphatidylinositol in the MPL diet. The SoyLec diet was, however, slightly higher in phosphatidylserine than the MPL diet. The chemical composition and the concentration of retinol and omega-3 fatty acids in the diets were the same. The concentration of astaxanthin was lower in serum of fish fed the MPL diet compared to the SoyLec diet and a larger proportion of astaxanthin metabolites, particularly idoxanthin, was found in serum, and all examined tissues in the MPL diet group. Genes involved in retinol synthesis were downregulated in both intestine and liver of fish fed low-marine diets, including in the diet with soy lecithin. In contrast, some genes in the retinol pathway were upregulated in fish fed the MPL diet, suggesting an increased synthesis of retinol from astaxanthin. The coincidence of this upregulation with increased idoxanthin levels in serum and tissues in the MPL group could suggest that reductive metabolism of astaxanthin is linked to retinol synthesis as suggested by Schiedt et al. [41]. This needs to be studied in more detail in future. The concentrations of retinol in the diets were not balanced in the present experiment, but the diets were supplemented with a vitamin premix that should cover the requirements for vitamin A. Rainbow trout fed diets low in vitamin A increased synthesis of vitamin A from astaxanthin [41], but effects of dietary retinol concentration within normal ranges on astaxanthin metabolism to retinol have not been studied in Atlantic salmon and how much of the digested astaxanthin that is converted to retinol it is not known.

Except for the MPL diet, the highest retention of astaxanthin was found in fish fed diets with plant protein. A possible explanation for the observed difference is that a larger fraction of astaxanthin is transported by albumin in the PP groups than in HDL and chylomicrons. The reduced expression of genes related to lipoprotein synthesis of chylomicrons and HDL in the intestine of fish fed the PP diets support this hypothesis. The albumin-bound astaxanthin may be transported directly into muscle from the blood, while astaxanthin bound in lipoproteins may perhaps to a greater extent be taken up by the liver and metabolized to other carotenoid or colorless compounds. The fat and astaxanthin concentrations in liver were not correlated. The lowest level of carotenoids in liver was found in fish fed the PP/PO diet and the highest in fish fed the MPL diet. However, both diet groups had high fat levels in liver, but only the MPL diet group had a normal intestine without fat accumulation. Thus, the accumulation of astaxanthin in the liver occurs under the condition of normal transport of lipids from the intestine. In a previous study by Ytrestøyl and Bjerkeng [79], astaxanthin was injected into the salmon's abdominal cavity to bypass the intestine, and the uptake of astaxanthin in muscle increased linearly with increasing amount of astaxanthin injected without any signs of saturation of transport capacity in the blood. Most likely, astaxanthin was bound to serum proteins such as albumin in the blood before uptake into muscle, and the transport capacity into the muscle does not seem to be limiting for muscle pigmentation since a highly colored fillet was achieved in only 8 weeks. Thus, shifts in transport mechanism may determine the fate of the astaxanthin, whether it is metabolized in the liver or passed on to the muscle and deposited.

Temperature may affect pigmentation of salmon through effects on digestibility and growth [36, 37, 46, 80]. There may also be interactions between diet composition and temperature on utilization of astaxanthin, particularly if dietary fatty acid composition is different [70]. Digestibility may not be affected at higher temperatures in diets with a high content of saturated fatty acids, but effects may be evident at lower temperatures when solubility of saturated fats is reduced. In the present study, the fatty acid composition of the diets was defined mainly by oil source. The diets with FO had a high content of short-chain SFAs and long-chain N-3 fatty acids, whereas diets with rapeseed oil had a high content of N-6 and N-9 fatty acids. Three diets were tested at high and low temperature, the marine diet (FM/FO), FM/PO, and PP/FO. There was no significant effect of temperature on digestibility. Fish at 6°C had lower feed intake compared to fish at 12°C, and thus a reduced passage route through the intestine, and more time available for absorption of astaxanthin. A higher feed intake has been shown to reduce the digestibility of astaxanthin [37], but when fish at different temperatures are fed the same amount of feed, lower temperature had a negative effect on the digestibility of astaxanthin [36]. There are not many published studies on the effect of temperature on pigment deposition efficiency in salmonids. No and Storebakken [81] did not find significant differences in flesh astaxanthin concentration in rainbow trout fed a marine-based diet at 5 and 15°C to similar BW, but the skin contained more carotenoids at 5°C. In contrast, improved flesh color was observed in Arctic charr reared at 8°C compared to at 12°C [38], and brighter fillet color in Artic charr raised at lower temperatures has also been reported by Gines et al. [39] and Imsland et. al. [40]. The different effects of temperature on pigmentation found in these studies may be due to differences between species. However, the studies on charr did not measure retention of astaxanthin, only the visual intensity of flesh color. The color intensity of the flesh is affected by the composition of the fillet, with lower color intensity at higher fillet fat content [82, 83]. Measuring pigment retention in the muscle is a more accurate method because it measures the actual deposition of the ingested amount of pigment.

In the present study, temperature affected the deposition of astaxanthin and metabolites in muscle, liver, and intestine. There were also interactions between diet composition and temperature, the retention of astaxanthin in the muscle was higher at 6°C than at 12°C for the FM/FO diet, whereas it was not affected by temperature in the FM/PO and PP/FO diets. Thus, at low water temperature, FO seems to improve astaxanthin deposition in the fillet compared to rapeseed oil. FM/FO diet had higher EPA + DHA concentration (4.1%) than the FM/PO diet (1.7%), and higher EPA and DHA have been shown to be positive for flesh deposition of astaxanthin [80]). Both the fat content and the concentration of idoxanthin in the liver were higher at 6° than at 12°C, and the increase was larger in the FM/PO diet than in the FM/FO diet. Lower temperature has previously been shown to increase liver fat content in Atlantic salmon, particularly in diets low in n-3 fatty acids [70, 74, 84]. The effect of EPA and DHA is probably linked to the critical role of these fatty acids in enhancing hepatic *β*-oxidation of fatty acids in [69, 85–87]. Astaxanthin is transported along with other lipids from intestine to liver, and the liver concentration of astaxanthin was also higher at lower temperature, and highest in the FM/FO group. However, the major difference between the FM/FO and FM/PO groups at 6°C was the much higher liver concentration of fat and idoxanthin in the latter group, even if the growth rate was similar for the diet groups. Thus, the fatty acid composition of the diet affected the liver astaxanthin deposition and metabolism to a larger extent at 6°C than at 12°C, similar to the fat deposition in liver. Low temperature has been shown to increase lipogenesis in liver in mammals and in fish [88, 89]. The concentration of some short-chain fatty acids was increased in liver at 6°C compared to at 12°C in all three diets in the present study, indicating that low temperature stimulate lipogenesis in salmon.

The effects of interactions between dietary fatty acid composition, PL content, and temperature on pigmentation have not been much studied. The diets used in commercial salmon farming have a low content of marine ingredients [2], and thus it is important to know how temperature affects astaxanthin utilization during the seawater production cycle with changing temperatures. The dietary requirement of PL in Atlantic salmon parr seems to be dependent on the water temperature, at 15°C the symptoms of steatosis were more severe than at 8° at similar dietary PL concentrations [65]. However, there are no studies on large fast-growing salmon in seawater that are exposed to variable temperatures during the seawater grow-out phase, and there is also large geographic variation in temperature profiles across the production area for Atlantic salmon. In the northern hemisphere, the retention of astaxanthin is known to change with season, with lower retention during winter when the temperature is low, and a drop in fillet color is often observed in the spring [80, 90, 91]. However, reduced astaxanthin during summer has also been found, and increased oxidative stress during a period with rapid growth was suggested to be the cause [92]. In the southern production areas like Tasmania, high summer temperatures are a problem, and reduced growth and discoloration of the fillet occur at water temperatures above 18–20°C [93, 94]. The discoloration was associated with an increase in metabolic products of carotenoids in liver and in plasma the retinoid metabolism pathway was elevated in pale fish compared to more colored fish. The changes in carotenoid content and metabolism were correlated with a reduction in PUFAs in pale fish and an increased biosynthesis of unsaturated fatty acids in fish with higher color [94]. Thus, there seem to be a link between the carotenoid and fatty acid metabolism, and diets with higher EPA and DHA content gave better fillet color and lower content of astaxanthin metabolites [5].

## 5. Conclusion

Sufficient dietary PL in low-marine diets is important for normal function of the intestine and digestibility of astaxanthin and fat. The diet with soy lecithin gave the best muscle pigmentation, whereas the diet with PL of marine origin (MPL) gave lower flesh pigmentation. However, the MPL diet was the only low-marine diet that had normal intestinal morphology with no sign of steatosis.

The importance of post-absorptive processes (metabolism in intestine and liver, transport from intestine to liver and muscle) for astaxanthin retention was demonstrated by the lower digestibility, but higher retention and lower metabolism of astaxanthin in the low-marine diet without added PL compared to the FM-based diets. The low-marine diets without added PL had severe steatosis in the intestine and reduced expression of genes involved in lipoprotein synthesis in the intestine, indicating reduced synthesis of chylomicrons and HDL. This could have favored transport of astaxanthin from the intestine directly to muscle rather than to the liver where astaxanthin is metabolized.

The results from this study imply that normal function of the intestine is optimal for growth but can result in less efficient pigmentation. Thus, adding PL to improve intestinal health and growth in salmon fed low-marine diets is necessary, but the PL source and diet concentration that will optimize pigmentation, intestinal health, and growth performance at different temperatures still needs to be determined. The improved retention at low temperature in a marine diet is interesting and should be investigated further, as it indicates that using marine ingredients, or higher levels of EPA + DHA and PL can be an advantage for pigmentation during cold water periods to improve fillet color and reduce liver lipid accumulation.

## Figures and Tables

**Table 1:** Diet composition

Ingredient (%)	Diet 1 FM/FO	Diet 2 FM/PO	Diet 3 PP/FO	Diet 4 PP/PO	Diet 5 MPL	Diet 6 SoyLec
Fishmeal	58.7	58.7	7.5	7.5	7.5	7.5
Wheat	13.5	13.5	10.0	10.0	10.0	10.0
Wheat gluten	—	—	22.5	22.5	22.5	22.5
Soy protein concentrate	—	—	26.0	26.0	26.0	26.0
Fish oil^a^	22.0	5.5	25.8	6.5	3.2	4.7
Rapeseed oil	—	16.5	—	19.4	19.4	19.4
Marine PL	—	—	—	—	3.2	—
Soy lecithin	—	—	—	—	—	1.7
Mineralmix	0.59	0.59	0.59	0.59	0.59	0.59
Vitaminmix	2.0	2.0	2.0	2.0	2.0	2.0
MSP*⁣*^*∗*^ (26% P)	2.5	2.5	2.5	2.5	2.5	2.5
Carop. Pink (10% Ast)	0.05	0.05	0.05	0.05	0.05	0.05
Yttrium oxide	0.01	0.01	0.01	0.01	0.01	0.01
Betafine	0.5	0.5	0.5	0.5	0.5	0.5
DL- Methionine	0.2	0.2	0.8	0.8	0.8	0.8
L-Lysine	—	—	1.7	1.7	1.7	1.7
Threonine	—	—	0.1	0.1	0.1	0.1

Abbreviations: FM, fishmeal; FO, fish oil; MPL, marinephospholipids; PO, plant oil; PP, plant protein; Soy Lec, soy lecitin.

^a^The amount of fish oil was adjusted to keep the diets similar in terms of fat, energy, and PL. The amounts of Soy lecithin and MPL in the diets were different due to different concentration of PL in the two products (based on analysis of the products before formulating the diets). The sum of fish oil and Soy lecithin and MPL + fish oil was 6.4% of the diet. The amount of rapeseed oil was kept constant at 19.4% in the low-marine diets. There were also small adjustments in FO in the other diets to achieve isoenergetic diets.

*⁣* ^*∗*^Monosodium phosphate

**Table 2:** Chemical composition of the diets.

Chemical composition		Diet 1 FM/FO	Diet 2 FM/PO	Diet 3 PP/FO	Diet 4 PP/PO	Diet 5 MPL	Diet 6 Soy Lec
Energy	(MJ/kg)	22.5	22.1	23.6	23.9	23.9	24.0
Fat	(%)	26.6	25.1	26.5	26.8	26.9	26.6
Nitrogen	(%)	6.7	6.7	7.0	6.9	7.0	7.1
Dry matter	(%)	94.7	93.2	95.7	95.2	95.6	95.2
Ash	(%)	12.2	12.3	5.5	5.4	5.6	5.7
Starch	(%)	9.0	9.1	10.5	10.5	10.2	10.0
Astaxanthin	(mg/kg)	41.5	41.3	45.8	45.1	48.3	49.7
Lutein	(mg/kg)	<1	2.2	<1	2.6	2.8	3.0
Zeaxanthin	(mg/kg)	<1	<1	<1	<1	<1	<1
Retinol	(IU/kg)	120 000	87 300	88 500	30 900	24 700	24 100
EPA + DHA	(%)	4.1	1.7	3.5	1.2	1.2	1.2
Phospholipid	(g/kg)	5.1	6.0	2.7	2.8	7.9	6.1

Abbreviations: FM, fish meal; FO, fish oil; MPL, marine phospholipids; PO, plant oil; PP, plant protein; Soy Lec, soy lecitin.

**Table 3:** Fish performance at 6 and 12 °C. Values are mean ± SEM (*N* = 3).

12°C	Diet 1 FM/FO	Diet 2 FM/PO	Diet 3 PP/FO	Diet 4 PP/PO	Diet 5 MPL	Diet 6 SoyLec
BW (g)	790 ± 28^a^	786 ± 34^a^	584 ± 19^c^	549 ± 22^c^	729 ± 24^ab^	704 ± 24^b^
SGR	1.49 ± 0.03^ab^	1.53 ± 0.03^a^	1.16 ± 0.02^c^	1.03 ± 0.03^c^	1.42 ± 0.01^b^	1.39 ± 0.04^b^
TGC	3.11 ± 0.08^a^	3.21 ± 0.09^a^	2.30 ± 9.03^c^	1.98 ± 0.08^c^	2.92 ± 0.04^b^	2.84 ± 0.07^b^
FCR	0.74 ± 0.0^ab^	0.76 ± 0.0^a^	0.70 ± 0.0^c^	0.70 ± 0.0^c^	0.72^bc^ ± 0.0	0.67^c^ ± 0.0
CF	1.27 ± 0.03^ab^	1.30 ± 0.03^a^	1.23 ± 0.02^b^	1.22 ± 0.01^b^	1.32 ± 0.03^a^	1.28 ± 0.01^ab^
HSI	1.22 ± 0.03^ab^	1.26 ± 0.04^a^	1.55 ± 0.05^c^	1.59 ± 0.07^c^	1.23 ± 0.05^a^	1.64 ± 0.06^c^
% fat in liver	5.4 ± 0.3^a^	10.7 ± 0.5^b^	15.3±0.0^c^	11.1 ± 0.3^b^	13.7 ± 1.1^bc^	14.2 ± 0.5^c^
Visceral fat score	3.1 ± 0.1^a^	3.0 ± 0.0^a^	2.0 ± 0.1^b^	1.7 ± 0.1^b^	2.8 ± 0.1^a^	2.2 ± 0.1^b^

**6°C**	**FM/FO**	**FM/PO**	**PP/FO**			

BW (g)	682 ± 14^a^	621 ± 23^a^	573 ± 13^b^	—	—	—
SGR	0.97 ± 0.01^a^	0.95 ± 0.03^a^	0.80 ± 0.01^b^	—	—	—
TGC	3.67 ± 0.02^a^	3.58 ± 0.13^a^	2.90 ± 0.07^b^	—	—	—
FCR	0.74 ± 0.00	0.77 ± 0.01	0.72 ± 0.02	—	—	—
CF	1.28 ± 0.02	1.26 ± 0.02	1.29 ± 0.03	—	—	—
HSI	1.50 ± 0.03^a^	1.54 ± 0.05^a^	1.78 ± 0.05^b^	—	—	—
% fat in liver	9.4 ± 0.3^b^	23.3 ± 0.5^a^	11.1 ± 0.0^b^	—	—	—
Visceral fat score	3.4 ± 0.2^a^	3.1 ± 0.5^a^	1.9 ± 0.1^b^	—	—	—

Abbreviations: BW, body weight; CF,condition factor; FCR, food conversion rate; FM, fish meal; FO, fish oil; HSI, hepatosomatic index; MPL, marine phospholipids; PO, plant oil; PP, plant protein; SGR, standard growth rate; Soy Lec, soy lecitin; TGC, thermal growth coefficient.

^a,b,c^ Mean values within a row with unlik esuperscript letters were significantly different (*P*< 0.05).

**Table 4:** Carotenoid concentrations (mg/kg) in serum, intestine, liver, and muscle at 12 °C and retention (% of eaten) of astaxanthin and total carotenoids in muscle.

(mg/kg)	Carotenoid	Diet 1 FM/FO	Diet 2 FM/PO	Diet 3 PP/FO	Diet 4 PP/PO	Diet 5 MPL	Diet 6 SoyLec
Serum	Astaxanthin	3.45 ± 0.38^a^	3.04 ± 0.64^a^	3.48 ± 0.10^a^	2.13 ± 0.21^b^	1.98 ± 0.26^b^	3.27±0.19^a^
	Idoxanthin	0.47 ± 0.12^b^	0.49 ± 0.14^b^	0.20 ± 0.02^c^	0.08 ± 0.02^c^	0.89 ± 0.07^a^	0.45 ± 0.06^b^
Intestine	Astaxanthin	1.90 ± 0.09	1.89 ± 0.18	2.29 ± 0.19	1.52 ± 0.13	2.11 ± 0.30	2.10 ± 0.15
	Idoxanthin	0.14 ± 0.06	0.18 ± 0.04	0.07 ± 0.01	0.05 ± 0.05	0.41 ± 0.05	0.18 ± 0.05
	Unidentified	0.13 ± 0.01	0.23 ± 0.03	0.13 ± 0.01	0.13 ± 0.02	0.23 ± 0.06	0.17 ± 0.03
Liver	Astaxanthin	1.57 ± 0.50^a^	1.45 ± 0.24^a^	1.42 ± 0.19^a^	0.57 ± 0.10^b^	1.30 ± 0.17^a^	1.16 ± 0.05^a^
	Idoxanthin	0.19 ± 0.08^bc^	0.32 ± 0.19^b^	0.09 ± 0.02^bc^	0.04 ± 0.02^c^	0.79 ± 0.03^a^	0.17 ± 0.05^bc^
	Unidentified	0.29 ± 0.09^a^	0.20 ± 0.03^b^	0.19 ± 0.01^b^	0.20 ± 0.05^b^	0.29 ± 0.04^a^	0.18 ± 0.04^b^
Muscle	Astaxanthin	2.29 ± 0.39^b^	2.35 ± 0.35^b^	2.77 ± 0.27^a^	2.39 ± 0.42^b^	1.91 ± 0.28^c^	3.08 ± 0.34^a^
	Idoxanthin	0.39 ± 0.06^c^	0.49 ± 0.07^b^	0.22 ± 0.04^e^	0.17 ± 0.03^e^	0.65 ± 0.11^a^	0.31 ± 0.09^d^
	Unidentified	0.02 ± 0.00^b^	0.05 ± 0.01^a^	0.03 ± 0.00^b^	0.03 ± 0.01^b^	0.03 ± 0.00^b^	0.03 ± 0.01^b^

**% Retention**							

Astaxanthin	—	5.9 ± 0.1^c^	5.7 ± 0.2^c^	7.7 ± 0.4^ab^	6.8 ± 0.6^b^	4.2 ± 0.1^d^	7.9 ± 0.7^a^
Carotenoids	—	6.9 ± 0.1^c^	7.0 ± 0.2^c^	8.4 ± 0.5^ab^	7.3 ± 0.7^b^	5.8 ± 0.1^d^	8.8 ± 0.4^a^

*Note:* Values are means per tank±SEM (N = 3). Different letters indicate significant differences (*p*< 0.05).

Abbreviations: FM, fish meal; FO, fish oil; MPL, marine phospholipids; PO, plant oil; PP, plant protein; Soy Lec, soy lecitin.

**Table 5:** Carotenoid concentrations (mg/kg) in intestine, liver, and muscle at 6 °C and retention (% of eaten) of astaxanthin and totalcarotenoids in muscle.

(mg/kg)	Carotenoid	Diet 1 FM/FO	Diet 2 FM/PO	Diet 3 PP/FO
Intestine	Astaxanthin	2.16 ± 0.08^b^	1.84 ± 0.07^c^	2.74 ± 0.09^a^
	Idoxanthin	0.27 ± 0.03^b^	0.51 ± 0.06^a^	0.14 ± 0.01^c^
	Unidentified	0.16 ± 0.01^b^	0.25 ± 0.01^a^	0.15 ± 0.01^b^
Liver	Astaxanthin	3.22 ± 0.49^a^	2.36 ± 0.31^b^	2.16 ± 0.31^b^
	Idoxanthin	0.96 ± 0.07^b^	1.72 ± 0.15^a^	0.28 ± 0.02^c^
	Unidentified	0.30 ± 0.03^a^	0.32 ± 0.03^a^	0.19 ± 0.01^b^
Muscle	Astaxanthin	2.78 ± 0.24^a^	2.18 ± 0.03^b^	2.92 ± 0.13^a^
	Idoxanthin	0.55 ± 0.04^b^	0.76 ± 0.07^a^	0.28 ± 0.02^c^
	Unidentified	0.03 ± 0.00^a^	0.05 ± 0.00^a^	0.02 ± 0.00^a^

**% Retention**				

Astaxanthin	—	7.2 ± 0.6^ab^	5.7 ± 0.0^b^	7.8 ± 0.5^a^
Carotenoids	—	8.8 ± 0.5^a^	7.8 ± 0.2^b^	8.7 ± 0.6^a^

*Note:* Values are means per tank±SEM (*N* = 3). Different letters indicate significant differences (*p*< 0.05).

Abbreviations: FM, fish meal; FO, fish oil; PO, plant oil; PP, plant protein.

## Data Availability

All data and information that are necessary for the reader to understand the results and draw the conclusions of the study can be found in tables and figures in the manuscript and in the Supporting Information.

## Supporting Information

Supporting Information Table S1: Fatty acid composition of the diets (% of total fatty acids). Fatty acids that were less than 1% of the total fatty acid content are not shown but are included in the total sum in the table. Table S2: Fatty acid composition in liver (% of total fatty acids) of Atlantic salmon fed the six experimental diets at 12°C and three diets at 6°C. Values are mean ± SD. N = 3. Fish meal (FM); fish oil (FO); plant oil (PO); plant protein (PP); marine phospholipids (MPL); soy lecitin (Soy lec). S3: Gene sequences of the genes shown in Figures 8 and 9.
