# Supplementary material for: Dietary Content of Plant Ingredients and Phospholipids Affects Astaxanthin Utilization and Lipid Deposition in Atlantic Salmon (Salmo salar L.)
Source: Aquac Nutr. 2025 Mar 21;2025:3454274. doi: 10.1155/anu/3454274 (PMC11952918; doi:10.1155/anu/3454274)
Supplement: Supporting Information — Table S1: Fatty acid composition of the diets (% of total fatty acids). Fatty acids that were less than 1% of the total fatty acid content are not shown but are included in the total sum in the table. Table S2: Fatty acid composition in liver (% of total fatty acids) of Atlantic salmon fed the six experimental diets at 12°C and three diets at 6°C. Values are mean ± SD. N = 3. Fish meal (FM); fish oil (FO); plant oil (PO); plant protein (PP); marine phospholipids (MPL); soy lecitin (Soy lec). S3: Gene sequences of the genes shown in Figures 8 and 9. [file 3454274.f1.zip › Supplement S1.docx]

Table S1: Fatty acid composition of the diets (% of total fatty acids). Fatty acids that were less than 1% of the total fatty acid content are not shown but are included in the total sum in the table.

|  | FM/FO | PP/PO | FM/PO | PP/FO | MPL | Soy lec |
| --- | --- | --- | --- | --- | --- | --- |
|  | (marine) | (low marine) | |  |  |  |
| 14:0 | 6.70 | 1.86 | 2.31 | 7.19 | 1.33 | 1.21 |
| 16:0 | 12.19 | 6.87 | 7.87 | 12.16 | 7.24 | 6.70 |
| 18:0 | 1.48 | 1.62 | 1.70 | 1.32 | 1.74 | 1.69 |
|  |  |  |  |  |  |  |
| 16:1 n-7 | 5.06 | 1.50 | 1.98 | 5.19 | 1.21 | 1.06 |
| 18:1 n-11 | 1.52 | 0.37 | 0.47 | 1.61 | 0.21 | 0.23 |
| 18:1 n-9 | 6.54 | 43.18 | 39.18 | 5.30 | 45.52 | 44.39 |
| 18:1 n-7 | 1.72 | 2.82 | 2.88 | 1.45 | 2.95 | 2.81 |
| 20:1 n-11 | 4.15 | 1.13 | 1.26 | 4.48 | 0.76 | 0.76 |
| 20:1 n-9 | 11.09 | 3.91 | 4.50 | 11.57 | 2.82 | 2.88 |
| 22:1 n-11 | 16.18 | 4.31 | 5.24 | 16.92 | 2.69 | 2.76 |
|  |  |  |  |  |  |  |
| 18:2 n-6 | 2.00 | 15.92 | 13.11 | 3.30 | 16.68 | 18.72 |
| 18:3 n-3 | 1.37 | 7.50 | 6.53 | 1.45 | 7.73 | 7.80 |
| 20:5 n-3 | 8.60 | 2.25 | 3.14 | 8.56 | 1.98 | 1.96 |
| 22:5 n-3 | 0.87 | 0.21 | 0.36 | 0.86 | 0.22 | 0.21 |
| 22:6 n-3 | 9.45 | 2.34 | 4.09 | 8.49 | 3.12 | 3.09 |
|  |  |  |  |  |  |  |
| Sum N-0 | 21.40 | 11.55 | 13.07 | 21.44 | 11.72 | 10.84 |
| Sum N-3 | 22.46 | 12.73 | 14.74 | 20.69 | 13.49 | 13.39 |
| Sum N-6 | 3.54 | 16.43 | 13.74 | 4.84 | 17.11 | 19.16 |
| EPA+DHA | 18.05 | 4.58 | 7.23 | 17.05 | 5.10 | 5.05 |

Fish meal (FM); Fish oil (FO); Plant oil (PO); Plant protein (PP); Marine phospholipids (MPL); Soy lecitin (Soy lec).
