# Supplementary material for: Dietary Content of Plant Ingredients and Phospholipids Affects Astaxanthin Utilization and Lipid Deposition in Atlantic Salmon (Salmo salar L.)
Source: Aquac Nutr. 2025 Mar 21;2025:3454274. doi: 10.1155/anu/3454274 (PMC11952918; doi:10.1155/anu/3454274)
Supplement: Supporting Information — Table S1: Fatty acid composition of the diets (% of total fatty acids). Fatty acids that were less than 1% of the total fatty acid content are not shown but are included in the total sum in the table. Table S2: Fatty acid composition in liver (% of total fatty acids) of Atlantic salmon fed the six experimental diets at 12°C and three diets at 6°C. Values are mean ± SD. N = 3. Fish meal (FM); fish oil (FO); plant oil (PO); plant protein (PP); marine phospholipids (MPL); soy lecitin (Soy lec). S3: Gene sequences of the genes shown in Figures 8 and 9. [file 3454274.f1.zip › Supplement S2.docx]

Table S2: Fatty acid composition in liver ((% of total fatty acids) of Atlantic salmon fed the six experimental diets at 12°C and three diets at 6°C. Values are mean±SD. N=3. Fish meal (FM); Fish oil (FO); Plant oil (PO); Plant protein (PP); Marine phospholipids (MPL); Soy lecitin (Soy lec).

| **Diet** | **FM/FO** | **FM/PO** | **PP/FO** | **PP/PO** | **MPL** | **Soy lec** | **FM/FO** | **FM/PO** | **PP/FO** |  |  |  |  |
| --- | --- | --- | --- | --- | --- | --- | --- | --- | --- | --- | --- | --- | --- |
| **Temp** | **12 °C** | **12 °C** | **12 °C** | **12 °C** | **12 °C** | **12 °C** | **6 °C** | **6 °C** | **6 °C** | **diet** | **temp** |  | **diet*temp** |
| C 14:0 | 3.7±0.4 | 2.1±0.1 | 4.1±0.1 | 1.9±0.1 | 1.6±0.1 | 1.6±0.1 | 5.2±0.4 | 2.5±0.1 | 5.4±0.2 | *** | *** | 6°>12° | ** |
| C 14:1 n-5 | 0.2±0.0 | 0.1±0.0 | 0.3±0.0 | 0.1±0.0 | 0.1±0.0 | 0.1±0.0 | 0.3±0.0 | 0.1±0.0 | 0.3±0.0 | *** | ** | 6°>12° | * |
| C 15:0 | 0.3±0.0 | 0.1±0.0 | 0.3±0.0 | 0.1±0.0 | 0.1±0.0 | 0.1±0.0 | 0.3±0.0 | 0.1±0.0 | 0.3±0.0 | *** | * | 6°>12° | NS |
| C 16:0 | 14.0±0.3 | 9.6±0.6 | 11.6±0.1 | 7.4±0.6 | 8.7±0.8 | 7.7±0.1 | 13.4±0.6 | 8.3±0.3 | 13.5±1.3 | *** | NS |  | ** |
| C 16:1 n-9 | 0.6±0.1 | 1.0±0.0 | 0.8±0.0 | 2.0±0.4 | 1.4±0.1 | 1.9±0.2 | 0.6±0.0 | 0.9±0.0 | 0.8±0.1 | *** | NS |  | NS |
| C 16:1 n-7 | 4.3±0.6 | 3.0±0.4 | 5.8±0.2 | 2.2±0.2 | 2.3±0.1 | 2.2±0.3 | 5.8±0.1 | 3.1±0.1 | 7.2±0.5 | *** | ** | 6°>12° | * |
| C 17:0 | 0.3±0.0 | 0.1±0.0 | 0.2±0.0 | 0.3±0.1 | 0.2±0.0 | 0.2±0.2 | 0.2±0.0 | 0.2±0.0 | 0.2±0.0 | *** | NS |  | NS |
| C 16:2 n-6 | 0.4±0.1 | 0.2±0.0 | 0.6±0.0 | 0.1±0.0 | 0.1±0.0 | 0.1±0.0 | 0.4±0.2 | 0.2±0.0 | 0.6±0.0 | *** | NS |  | NS |
| C 17:1 n-7 | 0.2±0.0 | 0.2±0.0 | 0.3±0.2 | 0.1±0.0 | 0.2±0.0 | 0.1±0.0 | 0.3±0.1 | 0.2±0.0 | 0.3±0.0 | * | NS |  | NS |
| C 18:0 | 3.3±0.5 | 3.7±0.1 | 3.9±0.2 | 3.2±0.3 | 3.8±0.4 | 3.2±0.2 | 4.4±0.2 | 3.9±0.2 | 4.7±0.2 | * | ** | 6°>12° | (*) |
| C 18:1 n-11 | 5.1±0.2 | 2.8±0.1 | 5.2±0.1 | 1.8±0.3 | 1.4±0.1 | 1.3±0.2 | 6.9±0.2 | 2.8±0.2 | 5.1±0.1 | *** | *** | 6°>12° | *** |
| C 18:1 n-9 | 13.5±1.5 | 37.2±1.1 | 15.5±0.1 | 36.3±1.0 | 40.3±1.2 | 37.2±1.8 | 17.2±0.9 | 37.3±1.2 | 18.1±0.6 | *** | ** | 6°>12° | * |
| C 18:1 n-7 | 2.4±0.2 | 3.0±0.0 | 2.7±0.2 | 2.7±0.1 | 2.8±0.1 | 2.4±0.2 | 3.2±0.1 | 3.1±0.1 | 2.8±0.0 | ** | *** | 6°>12° | *** |
| C 18:2 n-6 | 2.0±0.2 | 10.2±0.2 | 3.9±0.0 | 12.9±0.9 | 13.5±0.5 | 16.2±0.7 | 1.7±0.0 | 11.4±0.4 | 3.5±0.4 | *** | NS |  | ** |
| C 18:3 n-6 | 0.2±0.2 | 0.4±0.0 | 0.0±0.0 | 0.9±0.1 | 0.7±0.1 | 1.0±0.0 | 0.2±0.3 | 0.3±0.0 | 0.1±0.1 | *** | NS |  | ** |
| C 18:3 n-3 | 1.0±0.2 | 3.8±0.2 | 1.4±0.1 | 3.5±0.2 | 3.9±0.3 | 4.2±0.1 | 1.0±0.1 | 5.1±0.4 | 1.3±0.2 | *** | ** | 6°>12° | ** |
| C 20:1 n-11 | 1.1±0.2 | 0.6±0.1 | 1.7±0.1 | 1.2±0.0 | 0.8±0.1 | 0.8±0.6 | 1.0±0.0 | 0.7±0.1 | 1.4±0.2 | *** | NS |  | NS |
| C 20:4 n-3 | 1.8±0.2 | 0.6±0.1 | 1.9±0.2 | 0.5±0.1 | 0.3±0.0 | 0.3±0.0 | 2.7±0.2 | 0.8±0.1 | 1.8±0.0 | *** | ** | 6°>12° | NS |
| C 20:1 n-9 | 6.4±0.7 | 4.1±0.3 | 5.7±0.1 | 3.2±0.2 | 3.5±0.2 | 3.0±0.1 | 6.7±0.4 | 3.6±0.3 | 5.2±0.2 | *** | NS |  | NS |
| C 20:1 n-7 | 0.3±0.0 | 0.2±0.0 | 0.3±0.0 | 0.2±0.7 | 0.2±0.0 | 0.2±0.1 | 0.3±0.0 | 0.2±0.0 | 0.1±0.0 | ** | * | 12°>6° | ** |
| C 20:2 n-6 | 0.5±0.1 | 1.5±0.1 | 0.7±0.1 | 1.9±0.0 | 1.9±0.2 | 2.1±0.1 | 0.4±0.0 | 1.7±0.0 | 0.6±0.1 | *** | NS |  | * |
| C 20:3 n-6 | 0.4±0.1 | 1.0±0.2 | 0.5±0.0 | 1.7±0.1 | 1.8±0.3 | 2.5±0.2 | 0.2±0.0 | 0.9±0.0 | 0.4±0.1 | *** | (*) | 12°>6° | NS |
| C 20:4 n-6 | 0.7±0.0 | 0.5±0.2 | 0.5±0.1 | 1.0±0.0 | 0.4±0.1 | 0.6±0.2 | 0.5±0.0 | 0.3±0.0 | 0.4±0.1 | ** | ** | 12°>6° | NS |
| C 20:3 n-3 | 0.4±0.1 | 0.7±0.1 | 0.4±0.0 | 0.6±0.0 | 0.6±0.1 | 0.6±0.1 | 0.3±0.1 | 0.9±0.1 | 0.3±0.1 | *** | NS |  | ** |
| C 22:1 n-7 | 2.3±0.2 | 1.4±0.0 | 4.1±0.2 | 1.2±0.0 | 1.2±0.2 | 1.4±0.2 | 3.1±0.1 | 2.4±0.4 | 3.6±0.4 | *** | ** | 6°>12° | ** |
| C 22:1 n-11 | 5.2±1.3 | 1.1±0.1 | 3.6±0.0 | 0.6±0.1 | 0.5±0.0 | 0.4±0.0 | 3.9±0.5 | 0.7±±0.1 | 3.1±0.3 | *** | * | 12°>6° | NS |
| C 22:1 n-9 | 0.8±0.1 | 0.4±0.0 | 0.8±0.4 | 0.4±0.0 | 0.3±0.0 | 0.3±0.1 | 0.8±0.1 | 0.4±0.1 | 0.6±0.1 | *** | * | 12°>6° | NS |
| C 20:5 n-3 | 7.0±0.3 | 2.7±0.3 | 9.6±0.0 | 2.9±0.5 | 1.8±0.3 | 2.2±0.3 | 5.2±0.3 | 2.0±0.4 | 6.5±0.8 | *** | *** | 12°>6° | ** |
| C 22:5 n-3 | 2.4±0.4 | 0.8±0.0 | 2.3±0.0 | 0.7±0.2 | 0.4±0.1 | 0.5±0.3 | 1.5±0.0 | 0.4±0.1 | 1.1±0.1 | *** | *** | 12°>6° | * |
| C 22:6 n-3 | 14.9±5.7 | 4.5±0.9 | 6.1±0.3 | 5.3±0.9 | 2.8±0.6 | 2.9±1.0 | 7.4±0.5 | 3.1±0.2 | 5.7±1.1 | ** | * | 12°>6° | (*) |
| EPA+DHA | 21.9±5.9 | 7.2±1.1 | 15.8±0.7 | 8.1±1.3 | 4.7±0.9 | 5.1±1.2 | 12.6±0.8 | 5.1±0.6 | 12.2±1.8 | *** | ** | 12°>6° | (*) |
| Sum N-3 | 27.5±5.9 | 13.6±1.2 | 21.8±0.7 | 14.3±1.4 | 10.7±0.7 | 11.7±2.0 | 18.2±0.6 | 12.9±1.0 | 17.0±2.2 | ** | ** | 12°>6° | (*) |
| Sum N-6 | 4.1±0.3 | 13.7±0.3 | 6.1±0.0 | 18.4±0.3 | 18.5±0.8 | 22.5±0.6 | 3.3±0.4 | 14.8±0.4 | 5.5±0.6 | *** | NS |  | ** |
| Sum N-0 | 21.6±1.2 | 15.7±0.7 | 20.0±0.0 | 13.3±0.9 | 14.8±1.4 | 13.0±0.3 | 23.5±0.8 | 15.0±0.4 | 24.1±1.7 | *** | ** | 12°>6° | ** |

*** = *p*<0.001, ** = *p*<0.01, * = *p*<0.05, (*) = *p*<0.1
